# High-precision micro-displacement sensor based on tunnel magneto-resistance effect

**DOI:** 10.1038/s41598-022-06965-3

**Published:** 2022-02-22

**Authors:** Xuhu Wang, Wang Li, Li Jin, Meimei Gong, Junqiang Wang, Yujie Zhong, Yi Ruan, Chunhong Guo, Chenguang Xin, Mengwei Li

**Affiliations:** 1 School of Instrument and Electronics, North University of China, Taiyuan, 030051 Shanxi China; 2 Academy for Advanced Interdisciplinary Research, North University of China, Taiyuan, 030051 Shanxi China; 3 Nantong Institute of Intelligent Opto-Mechatronics, North University of China, Nantong, 226000 Jiangsu China; 4 School of Information and Communication Engineering, North University of China, Taiyuan, 030051 Shanxi China

**Keywords:** Design, synthesis and processing, Design, synthesis and processing

## Abstract

A high-precision micro-displacement sensor based on tunnel magneto-resistance effect is reported.We designed and simulated magnetic characteristics of the sensor, and employed chip-level Au-In bonding to implement low-temperature assembly of the TMR devices. We employed the subdivision interpolation technique to enhance the resolution by translating the sine-cosine outputs of a TMR sensor into an output that varies linearly with the displacement. Simultaneously, using the multi-bridge circuit method to suppress external magnetic and geomagnetic interference. Experimental result shows that the micro-displacement sensor has a resolution of 800 nm, accuracy of 0.14$\%$ and a full-scale range of up to millimeter level. This work enables a high-performance displacement sensor, and provides a significant guide for the design of a micro-displacement sensor in practical applications.

## Introduction

Micro-displacement measurement with high resolution and accuracy has numerous potential applications, such as automotive^1^, robotic system^2^, positioning^3^ and medical applications^4^. Techniques based on capacitive^5^, optical^6–9^, as well as magnetic sensors^10–12^ are widely used in non-contact displacement sensing. The capacitive type is susceptible to electromagnetic interference, and parasitic capacitance and fringe effects have been the major issues to improve the performance of the sensor^13^. Optical type is commonly used in displacement sensing due to its high accuracy and resolution, but it’s expensive and is not suited in dusty or harsh environments. Since magnetic field is not sensitive to the presence of nonconductive contaminants (such as oil, dirt, dust, etc.), magnetic type has excellent performance in dirt immunity and durability in harsh environments except for lower power consumption and simple interfacing circuitries.

Based on the sensing mechanism, there are several schemes have been proposed for the magnetic displacement sensors. The magneto-inductive (MI) sensors measure the inductance of coils changed with the permeability of the core when the permanent magnet moves^14^. This kind of linear MI-type sensors with a range of tens of millimeters are available. The sensing element based on the Hall effect is used to measure the magnetic field of the permanent magnet, whose field has a predefined pattern as a function of the distance from the permanent magnet^15–17^. As a new alternative, the displacement can be measured by detecting the magnetic flux density, according to the linear relationship between the magnetic flux and the reciprocal of displacement^18–23^.

In this paper, a displacement sensor based on tunnel magneto-resistance (TMR) effect with sub-micrometer level resolution and millimeter level of operation range is demonstrated. We present the design, simulation, and fabrication of TMR-based displacement sensor. To avoid temperature failure of TMR devices during manufacturing, we employed chip-level Au-In bonding to implement low-temperature assembly of the TMR devices^24–29^. We innovatively exploit unique TMR devices arrangement to extract quadrature components, and interpolation circuit to conduct nonlinear A/D conversion, implementing high-resolution sensitivity of the displacement sensor. In addition, we also proposed to use a multi-bridge circuit to achieve suppression of the surrounding magnetic interference, thus enhancing the operation accuracy of the displacement sensor.The designed TMR displacement sensor provides high sensitivity while achieving a large operation range.

## Principle of TMR displacement sensor

**Figure 1:**
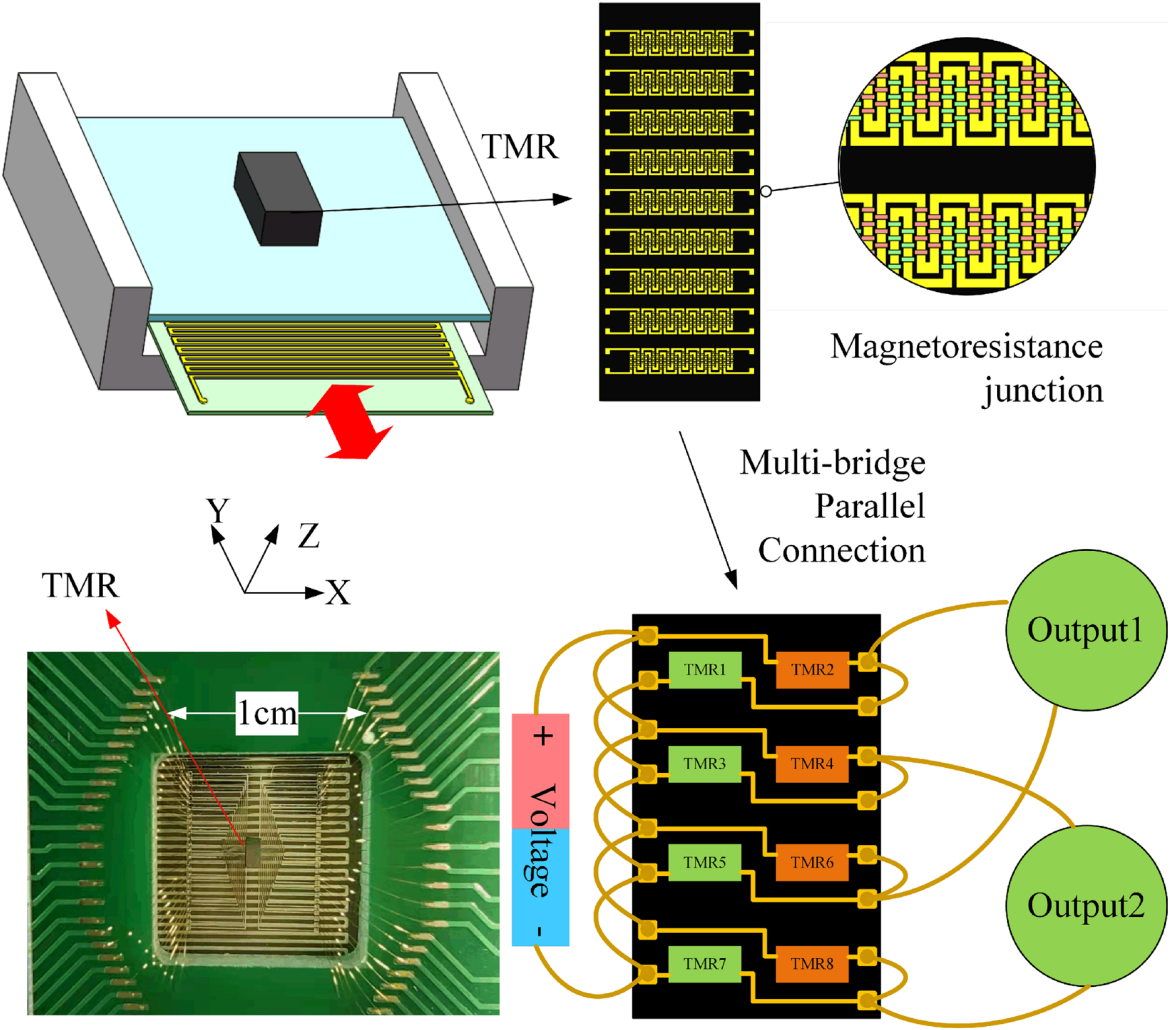
Schematic diagram of the TMR-based displacement sensor (Created by Microsoft Visio 2013 (15.0) and SOLIDWORKS(R) Premium 2016.).

A schematic diagram of the proposed displacement measurement system based on TMR effect is shown in Fig. 1. The TMR displacement sensor is composed of a copper electronic coil layer and TMR device layer. The electronic coil provides a uniform magnetic field in the X-axis direction and highly gradient magnetic field in the Y-axis (sensing) direction. The magnetic resistance structure consists of several groups of TMR devices, which has two kinds of magnetic resistance junctions with opposite polarity. It’s noteworthy that the magnetic field distribution and the sensitivity of the TMR devices directly decide the performance of TMR-based displacement sensor. During the operation of the proposed displacement sensor, it requires not only high gradient magnetic field in sensing direction, but also avoiding the electromagnetic interference. Thus, we employ a carefully designed electronic coil to provide the necessary magnetic field required to realize high sensitivity displacement measurements.

**Figure 2:**
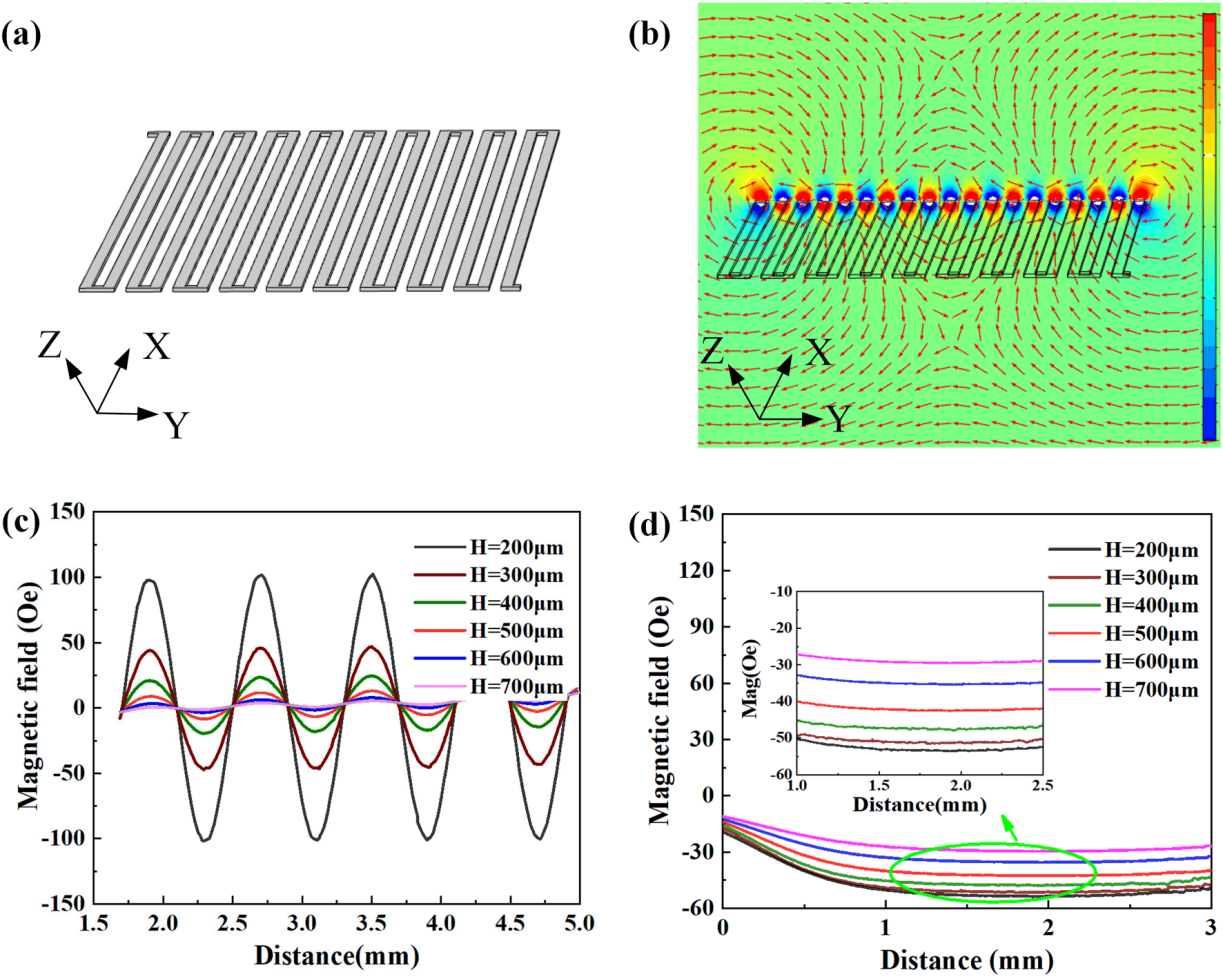
Magnetic field distribution analysis. *H* is the distance between electronic coil layer and TMR devices layer, ranging from 200 to $700\,\upmu \hbox {m}$ with a step of $100\,\upmu \hbox {m}$. (**a**) Model of the electronic coil (Created by Microsoft Visio 2013 (15.0) and SOLIDWORKS(R) Premium 2016.). (**b**) The magnetic field distribution along the sense direction. (**c**) The magnetic field along the sense direction (Y-axis) versus different layer distance *H*. (**d**) The magnetic field along the X-axis versus different layer height *H*.

To further verify the feasibility of the design, a finite element method (FEM) simulation is implemented by COMSOL Multi-physics. In order to analyze the magnetic field characteristics generated by the electronic coils, we set the dimensional parameters with the optimal width of 200 $\upmu$m, the thickness of 100 $\upmu$m, and the distance of 200 $\upmu$m between the adjacent coils, as shown in Fig. 2a. Figure 2b shows the finite element simulation of the magnetic field distribution along the sense direction. According to the simulation results, We further extract the magnetic field amplitude along Y-axis and X-axis versus different height *H* (ranging from 200 to 700 $\upmu$m with a step of 100 $\upmu$m), shown in Fig. 2c,d, respectively. From the simulation results, it can be seen that the magnetic field are basically symmetric and presents a quasi-sinusoidal distribution in the sense direction, with good consistency in the linear range, and the component of the magnetic field is almost a constant orthogonal to the sense direction. Moreover, the relationship between the magnetic field and the height *H* shows that the closer to the electronic coils, the greater the magnetic field strength.

Due to the extremely high sensitivity of magnetic sensing of the TMR devices, it can successfully apply in this displacement measurement system. However, the electromagnetic interference will degrade the performance during its operation. Here we employ the multi-bridge circuit method to suppress external magnetic and geomagnetic interference and avoid the integration error of the TMR devices effectively, as shown in Fig. 1. The differential voltage output of any two adjacent bridges in the multi-bridge circuits is^17^:

1 $$\begin{aligned} V= - \frac{KAV_0}{R_0}\sin {\frac{\pi (2y + d)}{2D}}\sin {\frac{\pi d}{2D}} \end{aligned}$$

where $V_0$ is the static voltage and $R_0$ is the static resistance of the TMR, *K* is the sensitivity of the TMR devices, *A* is the amplitude of magnetic feld, *D* is the line spacing of electric coils, *d* is the spacing distance of the adjacent bridge circuits and *y* is the displacement along the sense direction. From Eq. (1), two sinusoidal signals with a phase difference of 90$^{\circ }$ can be obtained by the movement of the electromagnetic coils which will cause magnetic field to change periodically. To further improve the sensitivity and the range of the displacement sensor, it’s necessary to apply relevant interpolation circuits.

## Fabrication processing

To implement the displacement sensor assembly, it’s necessary to connect the TMR devices to the supporting substrate via metal bonding. However, there exists a compromise between the shear strength and the bonding temperature due to the temperature failure of TMR devices. Due to the low-melting point of In, wafer-level Au-In bonding can be performed at considerably lower temperature than other metal eutectic bondings.

**Figure 3:**
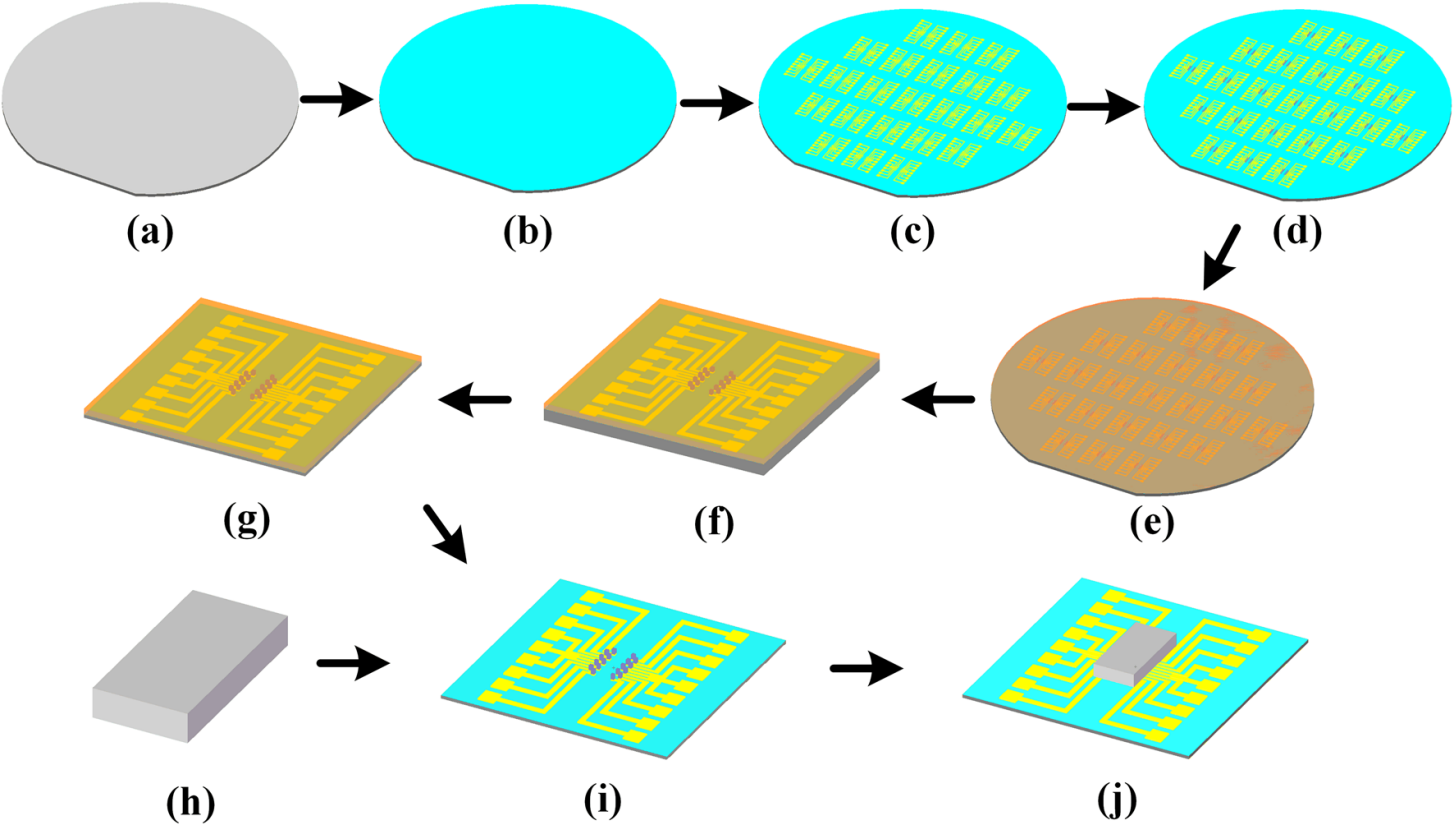
Fabrication processing flow of the bonding structure. (**a**) Sample wafer. (**b**) SiO$_2$ passivation layer was deposited on the surface by PECVD. (**c**) Sputtering of the electrode and bonding pads. (**d**) In layer evaporated onto the bonding pads. (**e**) Photoresist coated. (**f**) Scribing. (**g**) Reducing the thickness. (**h**) TMR device. (**i**) Photoresist removed. (**j**) Wafer-level Au-In bonding. (Created by Microsoft Visio 2013 (15.0) and SOLIDWORKS(R) Premium 2016.).

**Figure 4:**
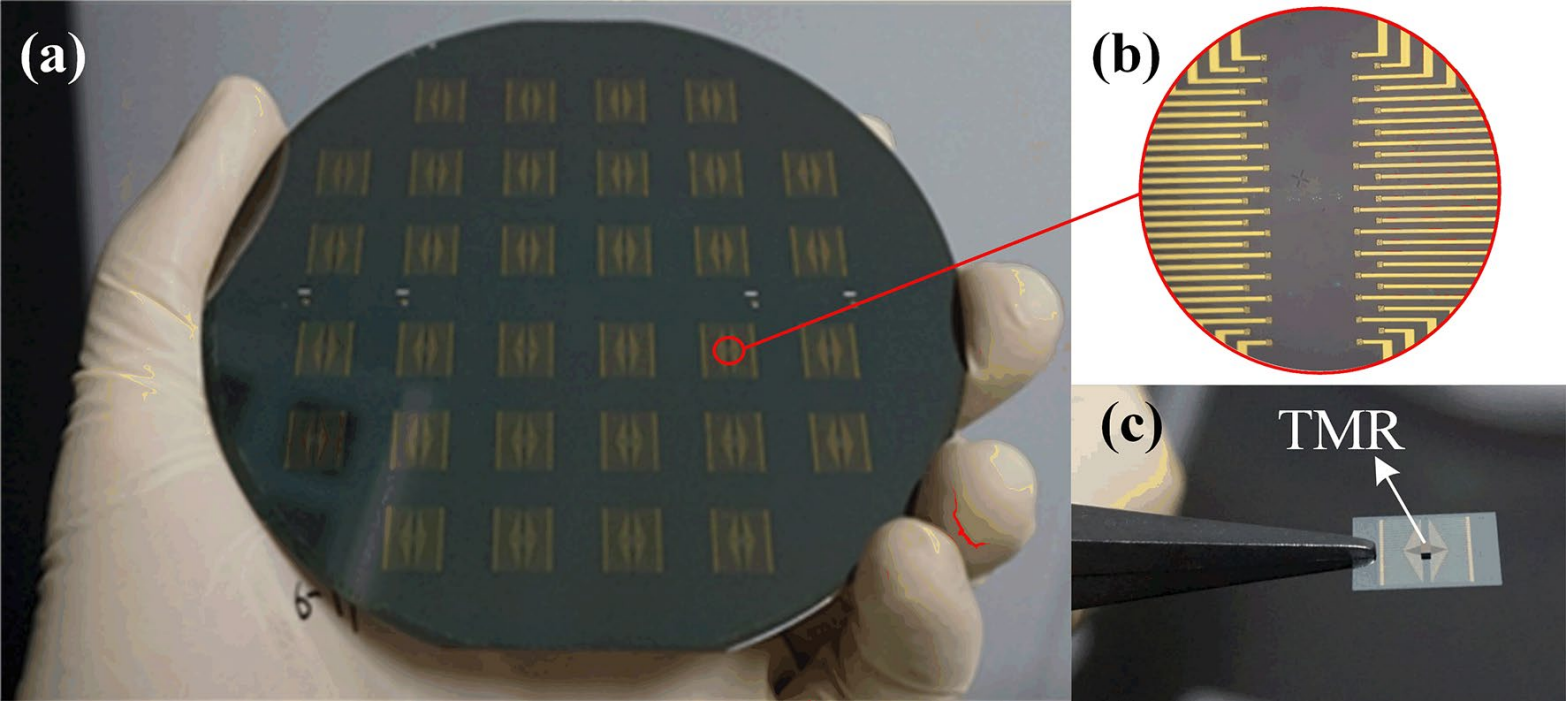
(**a**) Photograph of the fabricated 4-inch silicon wafer. (**b**) Micrograph of the bonding pads (Created by Microsoft Visio 2013 (15.0)). (**c**) Image of wafer-level Au-In bonding with TMR.

The fabrication process of the bonding structure is shown in Fig. 3a–j. A 100-nm-thick SiO$_2$ passivation layer was deposited on silicon wafer (Fig. 3a) surface by plasma-enhanced chemical vapor deposition (PECVD), as shown in Fig. 3b. A 10-nm-thick Cr adhesive layer and 200-nm-thick Au are deposited on the SiO$_2$ layer through magnetron sputtering. The multilayered-metal (Cr/Au) has been employed to form the electrode and bonding pads by lift-off technique (Fig. 3c). A 1.5 $\upmu$m thickness of In layer were evaporated onto the bonding pads (Fig. 3d) and then 4 $\upmu$m thickness of photoresist spin-coated on the bonding pads (Fig. 3e). With the protection of photoresist, we scribe the silicon wafer into pieces and reduce the thickness to 50 $\upmu$m (Fig. 3f–g). Finally, the alignment of the bonding die and the TMR devices (Fig. 3h) was performed to achieve the wafer-level Au-In bonding (Fig. 3j) after the removal of photoresist (Fig. 3i). The corresponding fabricated wafer is shown in Fig. 4a. A micrograph of the bonding pads and the image of wafer-level Au-In bonding with TMR are also shown in Fig. 4b,c, respectively.

To ensure there is pure In at the surface prior to bonding, the AuIn$_2$ growth should be limited by Au, and the required In/Au thickness ratio should be larger than the ratio corresponding to a complete conversion of all Au and In into AuIn$_2$, estimated to 3.1 by:

2 $$\begin{aligned} \frac{y(In)}{y_(Au)}= \frac{m(In) / \rho (In)}{m(Au) / \rho (Au)}=\frac{0.54/7.31}{0.46/19.3}= 3.1 \end{aligned}$$

where $m_{I_n}$ and $m_{A_u}$ are the mass of In and Au that are consumed into AuIn$_2$, 0.54 is the weight ratio of In in AuIn$_2$, 0.46 is the weight ratio of Au in AuIn$_2$,$\rho _{I_n}$ and $\rho _{I_n}$ are the densities of In and Au, respectively. Therefore, we deposited 1 $\upmu$m, 1.5 $\upmu$m, 2 $\upmu$m, 2.5 $\upmu$m In onto 0.2 $\upmu$m Au giving a In/Au ratio significantly larger than the requirement of 3.1.

**Figure 5:**
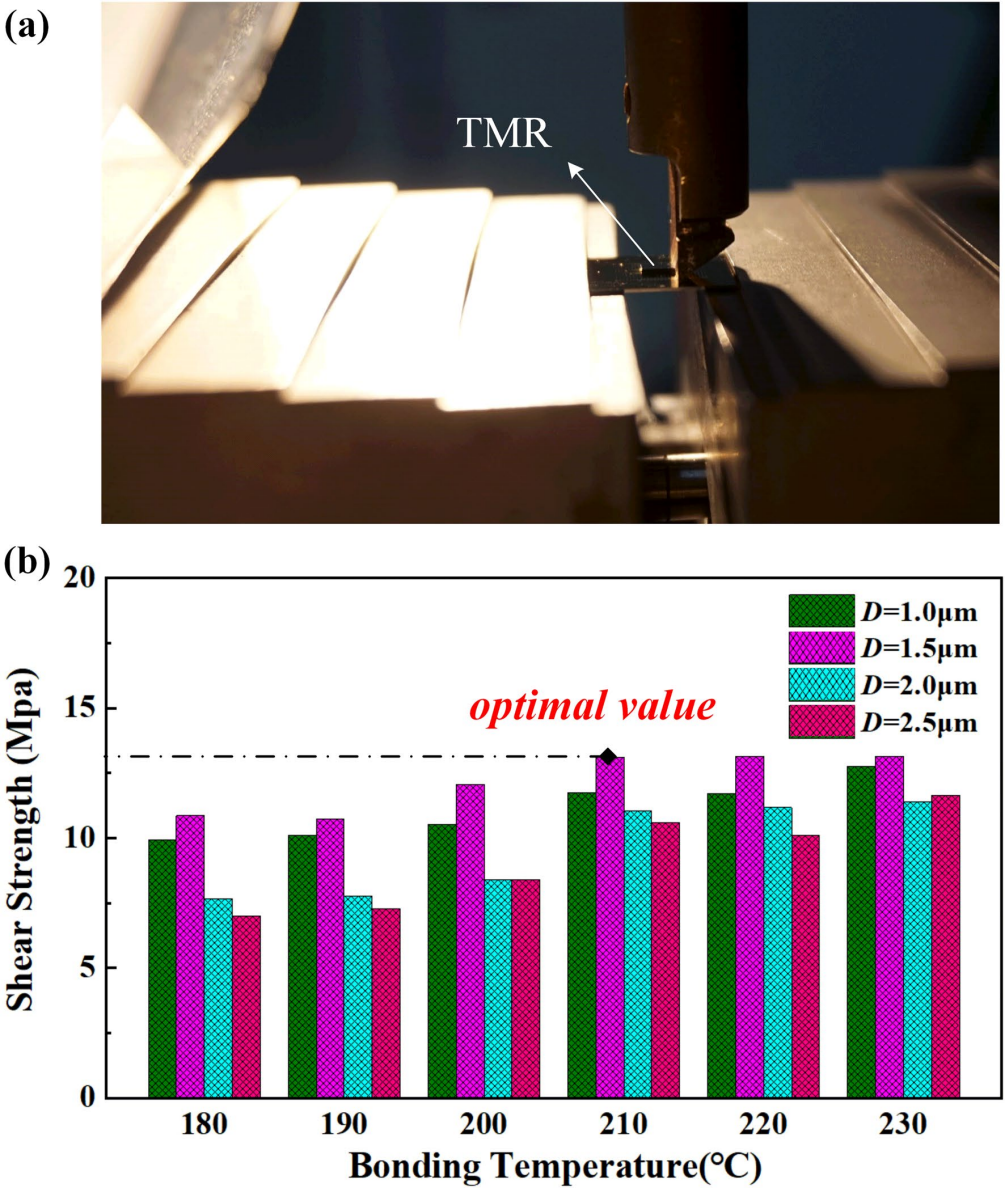
Shear test results. (**a**) Illustration of shear test configuration. (**b**) The shear strength as function of bonding temperature with different In thickness *D*.

Due to the TMR device is vulnerable to temperature, we analyze the bonding temperature that will affect the shear strength of Au-In bonding, as shown in Fig. 5. The schematic configuration of the shear test is shown in Fig. 5a. At a shear test pressure of 20 MPa and bonding time of 5 min, it’s obvious that the increment in shear strength is significant while the bonding temperature changes from 453 to 503 K (180–230 $^\circ \hbox {C}$). There exists an optimal In thickness, as shown in Fig. 5b, the shear strength of the bonding layer is up to more than 10 MPa with the thickness of 1.5 $\upmu$m. According to the remanence temperature coefficient, the magnetism of the magnetic material in the TMR decreases with increasing temperature which directly affects the TMR performance. It will mostly recover after cooling when the temperature rises within certain temperature range. It is experimentally demonstrated that the TMR will cause irreversible process when the temperature increases further more than 493 *K* $(220^\circ \hbox {C})$ and will damage as higher temperature, especially larger than 523 K (250 $^\circ \hbox {C})$. According to the above analysis of shear strength dependence, Au-In bonding experiment is performed at a lower temperature 483 *K* $(210^\circ \hbox {C})$ with the thickness of 1.5 $\upmu$m.

**Figure 6:**
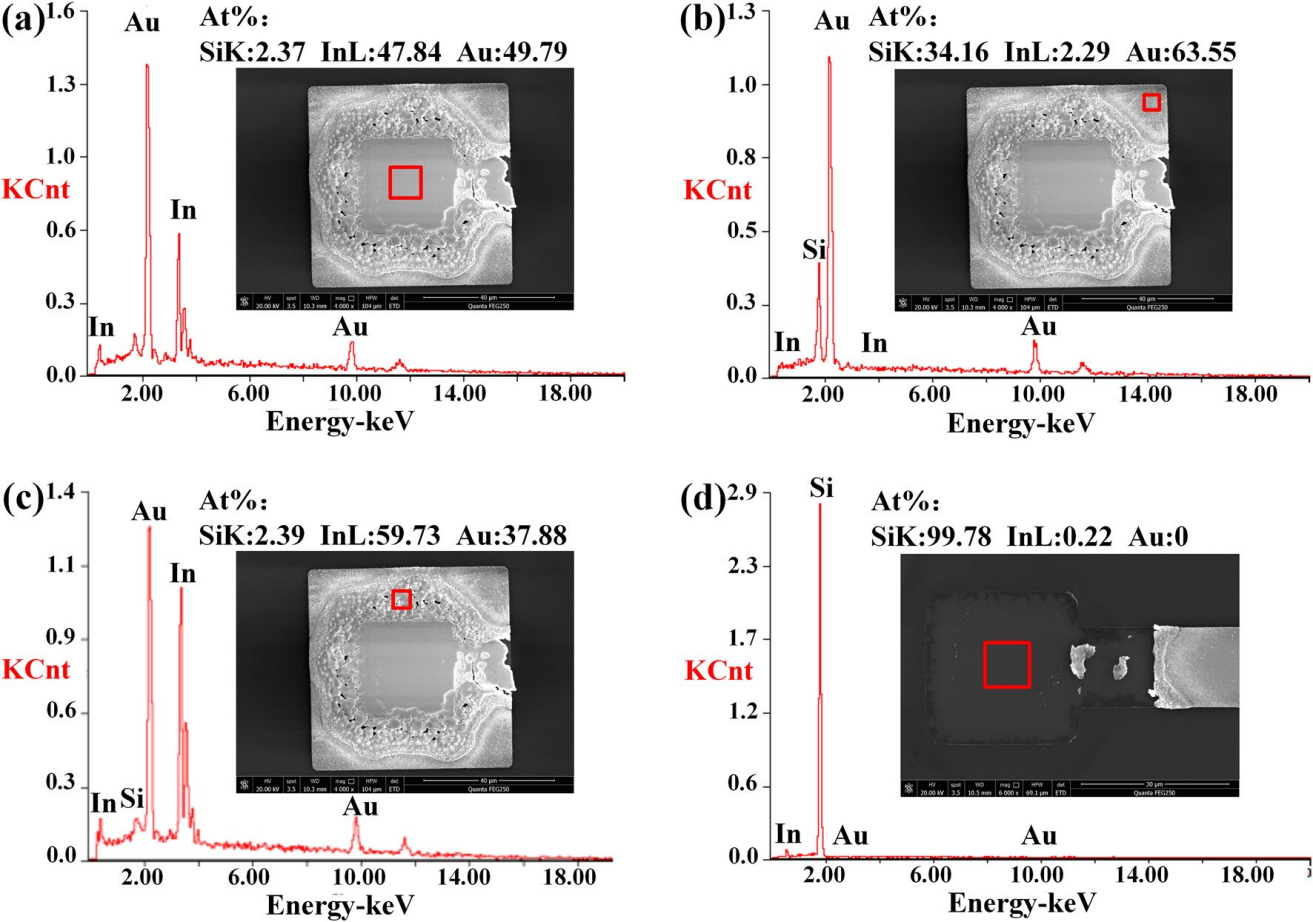
SEM/EDS image of fracture surface of bonded sample. Three different fracture positions were observed in (**a**–**c**), compared with the other fracture surface shown in (**d**), the fracture occurs at adhesive layer.

Figure 6 shows the SEM/EDS image of fracture surface of bonded sample. We observe three different positions in fracture surface, as shown in Fig. 6a–c. The positions of this fracture correspond to the bond interface between the substrate and the compound layer, indicating that rupture occurs at adhesive layer rather than cohesive fracture, compared with the other fracture surface shown in Fig. 6d. From the Fig. 6a,c, two Au-In intermetallic compounds (AuIn and AuIn$_2$) are present with In mole fraction 50 and 30 pct, demonstrating produced stable bonds in TMR fabrication process.

## Results and discussion

**Figure 7:**
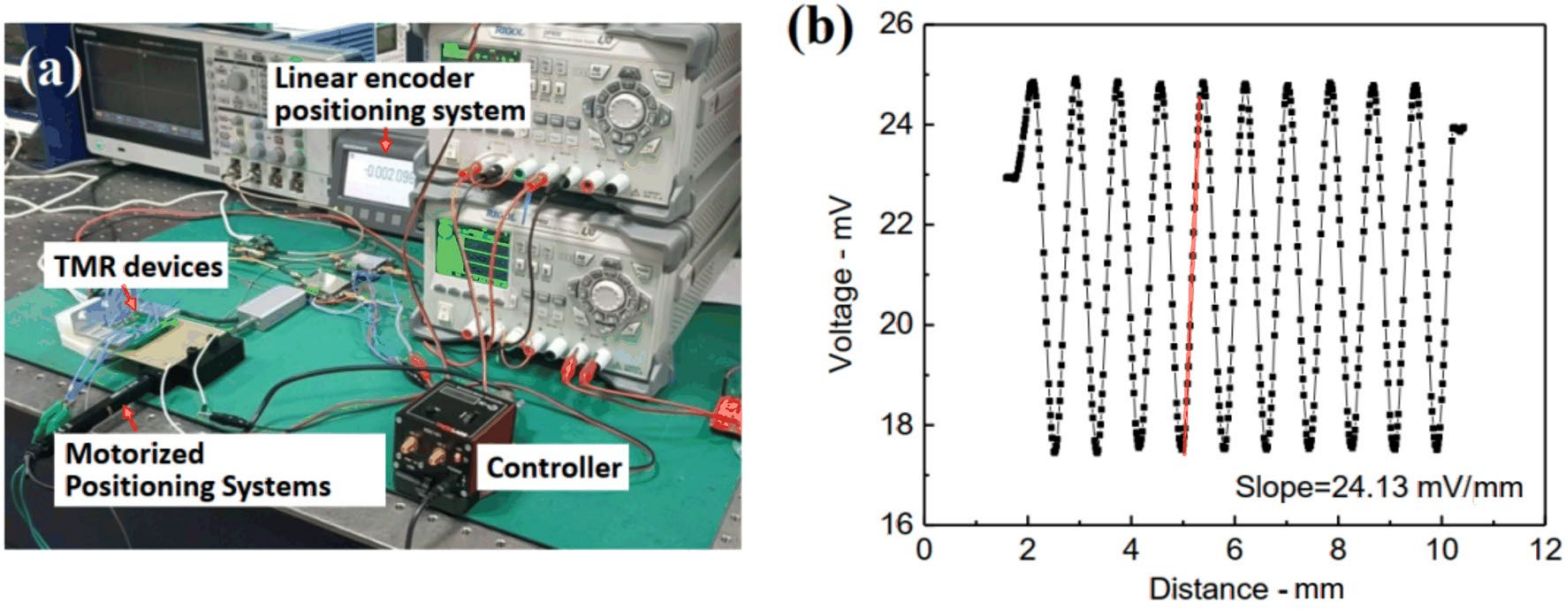
Experimental tests of the TMR devices. (**a**) Experimental setup. (**b**) The magnetic-resistance sensitivity of the TMR devices.

The displacement sensor behavior is not only dependent on the generated field but the sensitivity of TMR devices. It shows a linear relationship between the magneto-resistance and the external field, therefore, the influence of hysteresis phenomenon is negligible. To represent the characteristic of the TMR devices, we measure the magnetic-resistance sensitivity of the TMR devices with the operation voltage of 5 V, as shown in Fig. 7. The electric coil is fixed on the motorized positioning system, we record the output of the TMR device when moving the positioning system, as shown in Fig. 7a. According to the gradient of the magnetic field of 0.763 Oe/mm, the magnetic-resistance sensitivity of the TMR devices is 31.62 mV/Oe from the linear fitting of Fig. 7b.

**Figure 8:**
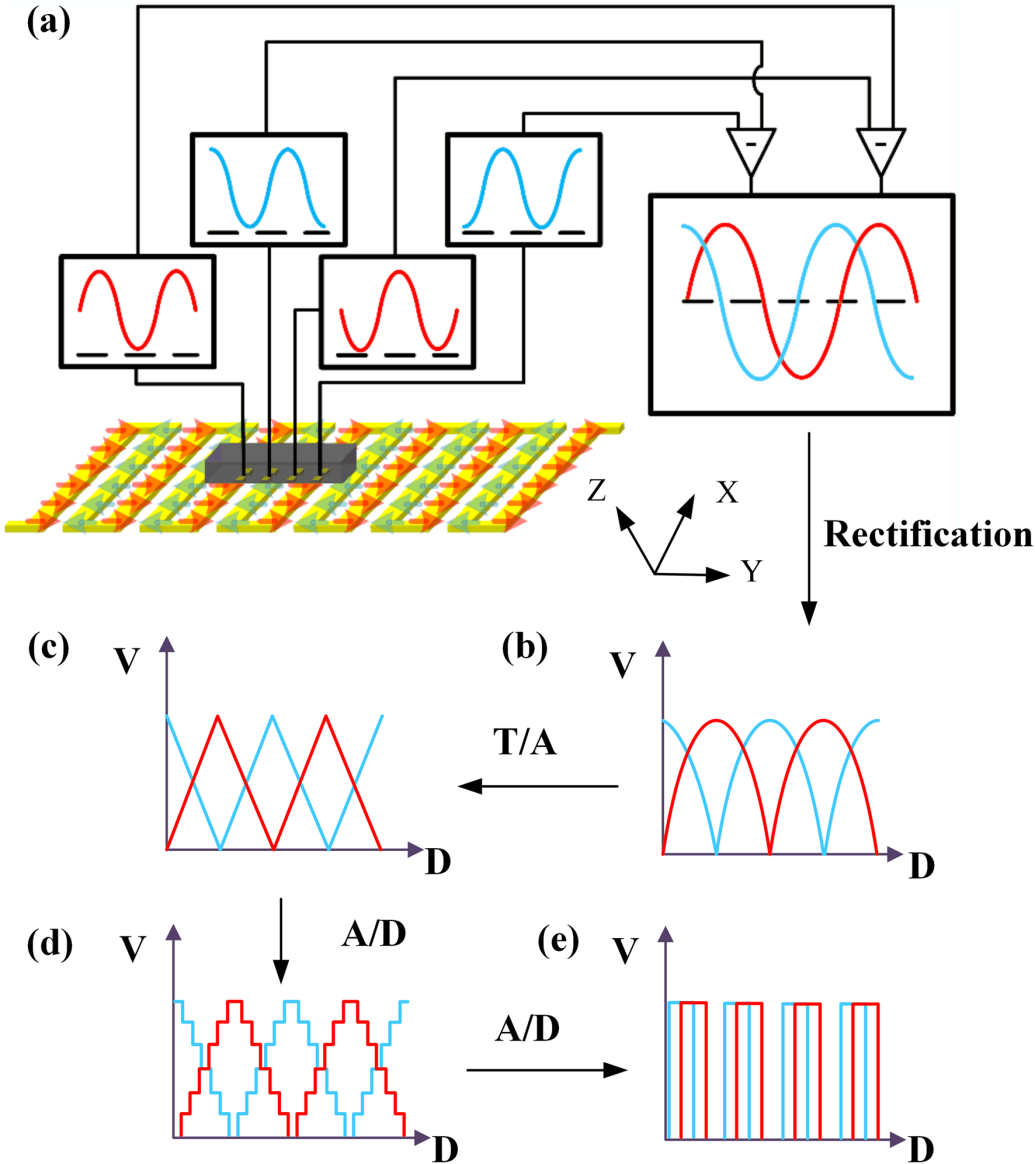
Principle of subdivision interpolation circuit. (**a**) A couple of sinusoids with phase difference of $90^\circ$(Created by Microsoft Visio 2013 (15.0) and SOLIDWORKS(R) Premium 2016.). (**b**–**d**) Process of the interpolation circuits converting the two sinusoidal signals into an industry standard incremental quadrature digital signal.

Due to the distribution of the magnetic field is a quasi-sine function curve in the sense direction, we arrange TMRs of the bridge circuits equidistant and the spacing distance is designed to a quarter of the period of the sinusoidal magnetic field. When the coils layer moves along the sense direction, two sinusoidal signals with a phase difference of 90$^{\circ }$ can be obtained from the output port. To enhance higher resolution of the displacement sensor, the interpolation circuit is employed to process the two sinusoidal signals to an industry standard incremental quadrature digital signal, as shown in Fig. 8. Here, we employed IC-TW8 chip from IC-Haus to interpolate the output signals. According to the dimensional parameters of electric coils, the output signal has a period of 800 $\upmu$m, the resolution of displacement sensor can reach to 800 nm after 1000 times subdivision^30,31^.

**Figure 9:**
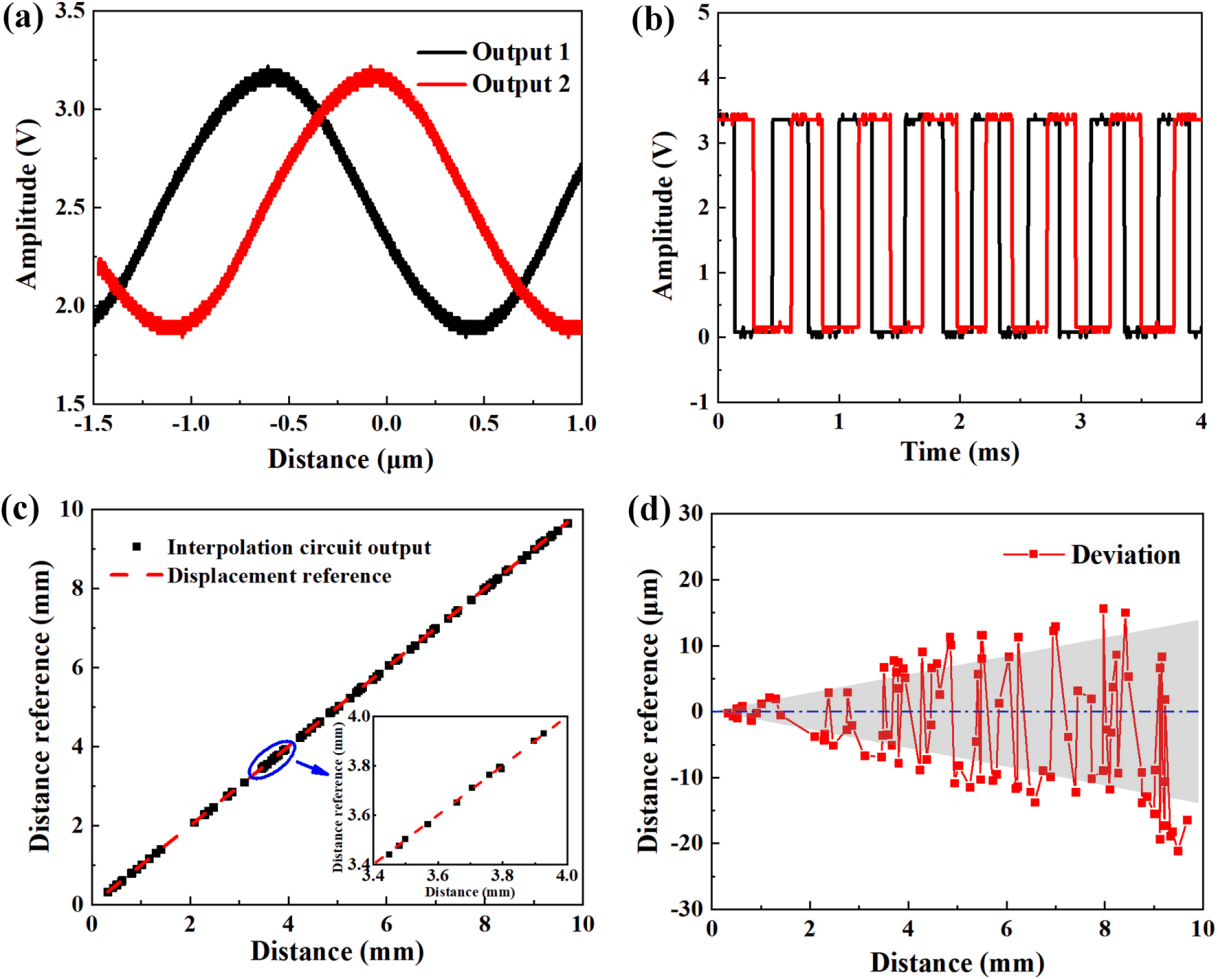
(**a**) Two sinusoidal signals with quadrature phase shift. (**b**) Standard incremental AB quadrature digital signal. (**c**) Comparison of the linear encoder positioning system and displacement output of the interpolation circuit. (**d**) Error distribution curve.

Figure 9a shows the input signal of the interpolation circuit with a digital oscilloscope, the red and black curves are quadrature signals with a phase difference of $90^\circ$. The red and black square wave signals are the relevant output of the interpolation circuit, which can accurately output the incremental AB quadrature digital signal, as shown in Fig. 9b. By comparing different interpolation factors, the final interpolation factor *N* is chosen to be 1000. Due to the factors (such as the temperature drift of the operational amplifier, DC offset error and gain error) will directly affect the accuracy of interpolation circuit, we employ the linear encoder positioning system produced by Heidenhain (Heidenhain MT1281, with the resolution of 0.5 nm and accuracy of $\pm 0.2 \upmu$m) to move along the sliding guide and use it as a displacement reference. Figure 9c shows the comparison the output of linear encoder positioning system and the interpolation circuit. It shows that the result of the displacement measurement agrees well with the reference data. From the error distribution curve, the average error values are 0.14$\%$ by comparing the linear encoder positioning system and the displacement of the interpolation circuit output, as shown in shaded area of Fig. 9d.

Due to the maximum interpolation factor of the interpolation circuit is 10,000, it’s not the highest level achievable in our research group currently, the resolution can reach to nanometer level if possible in the near future. Meanwhile decreasing the period of the current coil and reducing the height of TMR devices can effectively improve the measurement resolution.

## Conclusion

We proposed a novel high-sensitivity micro-displacement sensor based on tunnel magneto-resistance effect. The simulations are implemented to determine the magnetic characteristics of the device and exhibit the magnetic-resistance sensitivity of 31.62 mV/Oe. We employed the $1000\times$ interpolation circuit and multi-bridge tunneling magneto-resistance detection to achieve the TMR-based displacement sensor with sub-micrometer level resolution and millimeter level of operation range. With the deepening of research, the sensitivity of the displacement sensor can be further enhanced by reducing the distance between electronic coil layer and TMR devices layer, increasing the interpolation factor and decreasing the duty cycle of the electric coils. This kind of TMR-based displacement sensor possesses the advantages of high resolution, low energy consumption and is suitable for various applications in the future.

## Data Availability

All data generated or analysed during this study are included in this published article.
